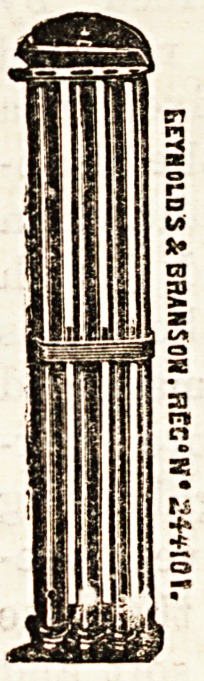# New Appliances and Things Medical

**Published:** 1895-06-29

**Authors:** 


### NEW APPLIANCES AND THINGS MEDICAL.

[We shall be glad to receive, at our Office, 428, Strand, London, W.C., from the manufacturers, specimens of all new preparations and appliances which may be brought out from time to time.]

### MARZA PORT WINE.

(Marza Company, 19 and 20, Wilson Street, Finsbury, E.C.)

The Marza wine, according to the proprietors, is made on a thoroughly scientific basis, and consists of a sound port wine to which coca, phosphorus, iron, and pepsine have been added. Mr. Lascelles Scott, in a report, states that he has also found a minute quantity of capsicine in the sample he examined. The proprietors claim it to be "the most scientific and palatable iron and coca wine," and state that they have been awarded a gold medal at Birmingham. Our analyst reports that he finds that the total extractive matter is higher than that found in an unmedicated port wine, pointing to the addition of the above ingredients, and that he has found an appreciable amount of iron in the ash. The quantity of phosphorus present as phosphoric acid is, however, not very large. He concludes that the wine is a valuable preparation, possessing valuable tonic properties, and believes that the presence of pepsine must render it most suitable for use at meal-time, as this ferment will beneficially aid digestion. The following figures show its composition :—

| | Parts in Percentages. |
|---|---|
| Alcohol by weight ... ... ... ... ... | 13·35 |
| Total extractive matter... ... ... ... | 13·08 |
| Mineral matter ... ... ... ... ... | 0·33 |
| Iron in ash ... ... ... ... | fair quantity |
| Phosphoric acid ... ... ... ... ... | 0·028 |

He further finds that the free volatile acidity calculated to acetic acid corresponds to 0·195 per cent., a quantity which shows that the port wine used in the manufacture is of sound quality. The fixed acidity calculated as tartaric acid is also low, amounting to 0·311. As a tonic wine the Marza port wine is to be recommended, and the fact that it has already been on the market for several years with yearly increasing sales shows that it is already appreciated by the public. We ought to add that although as shown by our analysis the wine is a medicated one, the taste and bouquet are very similar to a full-bodied port wine, so that its ingredients do not make the wine in the least unpalatable. On the contrary, Marza port is a pleasant wine and may be drunk with as much pleasure and enjoyment as the best port wine in the market.

### A PILL-BOX SHOOT.

(Messrs. Reynolds and Branson, 13, Briggate, Leeds.)

Not very long ago this firm produced a most useful contrivance in the shape of a bandage shoot, of which we gave an illustration at the time. They have again shown ingenuity in a time-saving apparatus which they have invented especially for use in dispensaries. The accompanying illustration explains the simple contrivance, whereby space and time is saved, and orderliness secured. The cost of the pill-box shoot is small, and the advantage is undoubted. It is made to hang on the walls, and is 37 inches long by 7 wide.

## Figures and Tables

**Figure f1:**